# Supplementary material for: A Critical View of the Application of the APEX Software (Aqueous Photochemistry of Environmentally-Occurring Xenobiotics) to Predict Photoreaction Kinetics in Surface Freshwaters
Source: Molecules. 2019 Dec 18;25(1):9. doi: 10.3390/molecules25010009 (PMC7017383; doi:10.3390/molecules25010009)
Supplement: Supplementary file 1 [file molecules-25-00009-s001.zip › 3.2.4_gcc-4.4.0.zip!/3.2.4_gcc-4.4.0/3.2.4_gcc-4.4.0/doc/octave/html/interpreter/Running-Octave-From-Within-Emacs.html]

Running Octave From Within Emacs - Untitled


Next: Using the Emacs Info Reader for Octave,
Previous: Using Octave Mode,
Up: Emacs Octave Support


---

### G.3 Running Octave From Within Emacs

The package octave provides commands for running an inferior
Octave process in a special Emacs buffer. Use

```
     M-x run-octave
```

to directly start an inferior Octave process. If Emacs does not know
about this command, add the line

```
     (autoload 'run-octave "octave-inf" nil t)
```

to your .emacs file.

This will start Octave in a special buffer the name of which is
specified by the variable `inferior-octave-buffer` and defaults to
`"*Inferior Octave*"`. From within this buffer, you can
interact with the inferior Octave process `as usual', i.e., by entering
Octave commands at the prompt. The buffer is in Inferior Octave mode,
which is derived from the standard Comint mode, a major mode for
interacting with an inferior interpreter. See the documentation for
`comint-mode` for more details, and use `C-h b` to find out
about available special keybindings.

You can also communicate with an inferior Octave process from within
files with Octave code (i.e., buffers in Octave mode), using the
following commands.

`C-c i l`: Send the current line to the inferior Octave process (`octave-send-line`). With positive prefix argument N, send that many lines. If `octave-send-line-auto-forward` is non-`nil`, go to the next unsent code line. `C-c i b`: Send the current block to the inferior Octave process (`octave-send-block`). `C-c i f`: Send the current function to the inferior Octave process (`octave-send-defun`). `C-c i r`: Send the region to the inferior Octave process (`octave-send-region`). `C-c i s`: Make sure that `inferior-octave-buffer' is displayed (`octave-show-process-buffer`). `C-c i h`: Delete all windows that display the inferior Octave buffer (`octave-hide-process-buffer`). `C-c i k`: Kill the inferior Octave process and its buffer (`octave-kill-process`).

The effect of the commands which send code to the Octave process can be
customized by the following variables.

`octave-send-echo-input`: Non-`nil` means echo input sent to the inferior Octave process. Default is `t`. `octave-send-show-buffer`: Non-`nil` means display the buffer running the Octave process after sending a command (but without selecting it). Default is `t`.

If you send code and there is no inferior Octave process yet, it will be
started automatically.

The startup of the inferior Octave process is highly customizable.
The variable `inferior-octave-startup-args` can be used for
specifying command lines arguments to be passed to Octave on startup
as a list of strings. For example, to suppress the startup message and
use `traditional' mode, set this to `'("-q" "--traditional")`.
You can also specify a startup file of Octave commands to be loaded on
startup; note that these commands will not produce any visible output
in the process buffer. Which file to use is controlled by the variable
`inferior-octave-startup-file`. If this is `nil`, the file
~/.emacs-octave is used if it exists.

And finally, `inferior-octave-mode-hook` is run after starting the
process and putting its buffer into Inferior Octave mode. Hence, if you
like the up and down arrow keys to behave in the interaction buffer as
in the shell, and you want this buffer to use nice colors, add

```
     (add-hook 'inferior-octave-mode-hook
               (lambda ()
                 (turn-on-font-lock)
                 (define-key inferior-octave-mode-map [up]
                   'comint-previous-input)
                 (define-key inferior-octave-mode-map [down]
                   'comint-next-input)))
```

to your .emacs file. You could also swap the roles of `C-a`
(`beginning-of-line`) and `C-c C-a` (`comint-bol`) using
this hook.
> **Note** that if you set your Octave prompts to something different
> from the defaults, make sure that `inferior-octave-prompt` matches
> them. Otherwise, *nothing* will work, because Emacs will not know
> when Octave is waiting for input, or done sending output.
